# “*Andrà tutto bene*”: Associations Between Character Strengths, Psychological Distress and Self-efficacy During Covid-19 Lockdown

**DOI:** 10.1007/s10902-020-00321-w

**Published:** 2020-10-13

**Authors:** Nicole Casali, Tommaso Feraco, Marta Ghisi, Chiara Meneghetti

**Affiliations:** 1 Department of General Psychology, University of Padova, via Venezia 8, Padua, Italy; 2 Pentathlon Srl, Napoli, Italy

**Keywords:** Character strengths, COVID-19, Psychological distress, Self-efficacy, Psychological impact, Lockdown

## Abstract

The Covid-19 pandemic obliged people around the world to stay home and self-isolate, with a number of negative psychological consequences. This study focuses on the protective role of character strengths in sustaining mental health and self-efficacy during lockdown. Data were collected from 944 Italian respondents (mean age = 37.24 years, SD = 14.50) by means of an online survey investigating character strengths, psychological distress and Covid-19-related self-efficacy one month after lockdown began. Using principal component analysis, four strengths factors were extracted, namely transcendence, interpersonal, openness and restraint. Regression models with second-order factors showed that transcendence strengths had a strong inverse association with psychological distress, and a positive association with self-efficacy. Regression models with single strengths identified hope, zest, prudence, love and forgiveness as the strengths most associated with distress, love and zest as the most related to self-efficacy and zest to general mental health. Openness factor and appreciation of beauty showed an unexpected direct relation with psychological distress. These results provide original evidence of the association of character strengths, and transcendence strengths in particular, with mental health and self-efficacy in a pandemic and are discussed within the field of positive psychology.

**Electronic supplementary material:**

The online version of this article (10.1007/s10902-020-00321-w) contains supplementary material, which is available to authorized users.

## Introduction

Research on the psychological impact of quarantine due to the Covid-19 pandemic has largely focused on estimating the prevalence of mental health disorders (e.g. anxiety and depression) in the general population (Brooks et al. 2020) and related socio-demographic risk factors. Very few psychological and clinical studies have examined individual characteristics that can support mental health under such circumstances (Asmundson and Taylor 2020). In addition to the mental health burden most often reported in the literature, there is some evidence of positive changes occurring after a pandemic, including an increase in compassion and empathy, post-traumatic growth, and self-empowerment (Chew et al. 2020). Positive psychology is increasingly addressing the scientific study of how positivity affects life struggles, and how life challenges can bring about positivity (Niemiec 2019). Character strengths (Peterson and Seligman 2004) are among the most investigated individual characteristics in the field of positive psychology. They are mainly general dispositions that have been repeatedly associated with well-being (Niemiec 2013), and resilient growth following events such as the September 11 attacks (Peterson and Seligman 2003), natural disasters (Duan and Guo 2015), shooting tragedies (Schueller et al. 2015), and the Paris terrorist attacks (Lamade et al. 2019). Therefore, character strengths can be seen as useful individual characteristics in protecting mental health (reducing symptoms of distress and increasing self-efficacy) following a pandemic too. The present study newly examines the influence of character strengths on mental health in the time of Covid-19.

### Evidence of the Psychological Impact of Covid-19

Literature on the psychological impact of the Covid-19 pandemic is on the rise. A review of its impact on mental health (Nobles et al. 2020) retrieved 6 published cross-sectional studies, all conducted on the Chinese general population (Cao et al. 2020; Li et al. 2020b, c; Liu et al. 2020; Wang et al. 2020a; Zhang and Ma 2020). The findings resemble those relating to previous pandemics (Brooks et al. 2020; Chew et al. 2020) with symptoms of anxiety, depression, and post-traumatic stress disorder (PTSD) being the most often reported in the general population. Several demographic variables were associated with worse outcomes, including: female gender; living in Wuhan and Hubei province (the hardest-hit area); being a student; having relatives infected; job loss due to the pandemic; and pre-existing physical health issues (e.g. chronic disease). Similar results emerged in large community samples (n = 7236, Huang and Zhao 2020), and in countries other than China (Iran, Moghanibashi-Mansourieh 2020; India, Roy et al. 2020; Italy, Cellini et al. 2020; Moccia et al. 2020; North-Spain, Dosil-Santamaria et al. 2020; Paraguay, Rios-González and Palacios, 2020; UK, Shevlin et al. 2020). Furthermore, a 4-week longitudinal study (Wang et al. 2020b) focusing on PTSD symptoms and psychological distress (n = 478) showed a significant decline in PTSD symptoms over time, though they remained above cut-off, while no significant changes were seen in depression, anxiety and stress levels. In other words, the psychological impact of Covid-19 may persist, as already seen in the case of the SARS pandemic in survivors (Mak et al. 2009), in the general population (Yeung and Fung 2007), and in university students (Cheng and Cheung 2005; Qian et al. 2005). Some evidence has also emerged regarding the protective factors, which include: living in urban areas; living with parents; having a steady family income (Cao et al. 2020); moderate physical exercise (Zhang et al. 2020); both secure and avoidant attachment styles (Moccia et al. 2020); confidence in doctors; perceived likelihood of surviving and low risk of contracting Covid-19; and satisfaction with health information and personal precautionary measures (Wang et al. 2020a). Interestingly, a study conducted in 31 Chinese regions (Li et al. 2020a) found that a low self-control (in the sense of not being able to adopt an appropriate behavior) was associated with a greater perceived dangerousness of Covid-19 and more mental health problems. The present study is one of the few to focus on the importance of individual personality traits for mental health during the Covid-19 lockdown.

### Character Strengths and Adversity: Relationships with Distress and Self-efficacy

In their seminal work, Peterson and Seligman (2004) conceptualize character strengths as “the psychological ingredients—processes or mechanisms—that define the virtues” (p. 13). They identify six core virtues, also called “the high six”, i.e. wisdom, courage, humanity, justice, temperance, and transcendence that have been recognized across many different cultures and historical periods. To qualify as such—and distinguish from other positive traits—they must meet 10 criteria (e.g. contribute to one’s and others’ fulfilment, be morally valued per se, not diminish others, not have an opposite leading to positive outcomes, be assessable and trait-like). Peterson and Seligman (2004) then developed a tool for measuring strengths (the Values in Action Inventory of Strengths-240; VIA-IS-240), and identified, by factor analysis, five factors closely mirroring the six core virtues, which they named emotional, intellectual, interpersonal, restraint and theological. Since then, numerous studies have examined the internal structure of the VIA-IS-240, and the relationship between the factors identified and variables of interest (e.g. Martínez-Martí and Ruch 2017; Petkari and Ortiz-Tallo 2018; Weber et al. 2013). This type of analysis, combined with a close examination of the role played by each single strength, may enable a given character strength to be linked to a specific factor, as if they were virtues (i.e. by seeing strengths as psychological mechanisms leading to factors), and the number of variables to consider to be reduced.

Character strengths have long been considered among the personal assets that support well-being, particularly in challenging life situations. While their primary role is to contribute to the fulfilment of the individual and the achievement of a good life, “strengths and virtues determine how an individual copes with adversity” (Peterson and Seligman 2004, p. 17).

From a theoretical standpoint, Niemiec (2019) recently pointed out that character strengths have three “adversity functions”, enabling individuals to thrive when times are hard: buffering—character strengths use can prevent problems (prior to adversity); reappraisal—character strengths can help explain or reinterpret problems (during adversity); and resilience—character strengths support recovery (after adversity).

As regards the buffering function, transcendence/theological (e.g. hope, spirituality) and restraint strengths (e.g. perseverance, self-regulation) were found to predict resilience over and above factors such as positive affect, self-efficacy, optimism, social support, self-esteem, life satisfaction, and sociodemographic variables (Martínez-Martí and Ruch 2017). All character strength factors (except for transcendence strengths) also showed positive associations with general self-efficacy. All these factors (except for interpersonal strengths, including humility, fairness and teamwork as single character strengths) were also significant predictors of general self-efficacy beliefs in Israeli adolescents (Weber et al. 2013); and self-efficacy mediated the relationship between emotional strengths and life satisfaction. More specifically, Ruch et al. (2014) found that, among children and adolescents, the strengths showing the strongest associations with general self-efficacy were hope, zest, gratitude, perspective, creativity, social intelligence and teamwork (i.e. mainly transcendence and emotional strengths). Moreover, restraint and interpersonal strengths have been found negatively associated with depression and anxiety in college students reporting stressful situations (Duan 2016). Transcendence strengths like hope, zest and love, seem to have a particularly strong relationship with mental health outcomes, such as depression (Huta and Hawley 2010), quality of life (Smedema 2020), and life satisfaction (Bruna et al. 2019; Martínez-Martí and Ruch 2014).

Concerning the reappraisal function, some authors (Duan and Wang 2018; Haridas et al. 2017) identified different profiles of individuals based on their character strengths, and found these profiles associated with different levels of psychological distress. For instance, a latent profile analysis conducted by Duan and Wang (2018) distinguished their sample between “at-strengths” and “at-risk” individuals, who respectively exhibited high and low levels of restraint, and intellectual and interpersonal strengths. The “at-strengths” group experienced higher levels of psychological well-being and lower levels of psychological distress than the “at-risk” group.

Interpersonal, restraint and transcendence strengths also correlated negatively with general psychological distress in adolescents exposed at length to war, political conflict, and terrorism in Israel (Shoshani and Slone 2016), thus supporting the resilience function of character strengths.

In short, character strengths are personal features fundamental to well-being and life satisfaction but may be particularly important in times of adversity. They could therefore have a fundamental role in a pandemic lockdown too.

### The Present Study

In the aftermath of severe Covid-19-related restrictions, Italian people’s personal and working lives have undergone necessary, drastic changes. Italy was the first Western country to experience the dramatic effects of Covid-19, and it has been among the countries hardest hit by the pandemic (World Health Organization, WHO 2020). According to the Italian Higher Health Institute (HHS 2020), by April 28th 2020 (when our data collection ended), there had been nearly 200,000 people infected, and more than 25,000 had died. It is important to study the role of positive individual characteristics in such a situation to clarify whether certain personal resources are associated with the negative impact of lockdown and the dramatic health care situation of a pandemic, suggesting their importance in yet another life situation.

Two early studies in Italy, conducted respectively two weeks and one month after the lockdown was imposed in early March 2020 (Cellini et al. 2020; Moccia et al. 2020), identified from moderate to extreme levels of psychological distress in quite a large proportion of participants (18.6% in Moccia et al., 24.2–50.2% in Cellini et al.). The present study examines the individual features potentially helping individuals in the Italian general population to cope after a month in quarantine, given the reports of negative effects of such situations on mental health (e.g. anxiety and depression) persist over time (Wang et al. 2020b). More specifically, the main goal of our study was to analyze whether and which character strengths are associated with people’s mental health and Covid-19-related self-efficacy. Such a relation may rely on the contribution of strengths in favoring individuals’ ability to contain their psychological distress when facing adversity.

With this aim, we examined factor composition and the relation of both second-order and single character strengths with psychological distress and self-efficacy outcomes. In line with previous reports on populations experiencing stress in various situations (e.g. university life, Martínez-Martí and Ruch 2017; Duan 2016; political conflict and war, Shoshani and Slone 2016; Weber et al. 2013), we expect transcendence and restraint to be the second-order strengths most strongly associated with psychological distress and self-efficacy. It has repeatedly been suggested that these strengths are particularly important in giving individuals the energy and determination to face challenging situations, and helping them to regulate their behavior under stressful circumstances. We therefore hypothesize that transcendence and restraint second-order strengths may relate to lower levels of psychological distress (i.e. depression, anxiety and stress), and higher levels of Covid-19-related self-efficacy. Analyzing single strengths will also enable us to see which of them has a specific relation with the dependent variables. We might expect strengths such as hope, love, gratitude and zest (usually part of the transcendence strengths factor) to have a detectable influence in a pandemic, as previously reported in non-pandemic settings, on both mental health (Huta and Hawley 2010; Petkari and Ortiz-Tallo 2018) and self-efficacy (Ruch et al. 2014).

## Materials and Methods

### Participants

The study included Italian native speakers at least 18 years of age and living in Italy. All participants took part in the study voluntarily and approved the consent form before taking part. The study was approved by the Ethical Committee of the University of Padova (n. 3531). Data collection lasted 3 weeks, from April 7th (one month after lockdown was officially declared) to April 28th, 2020. Starting on March 9th, 2020, the nationwide lockdown measures made it impossible to travel anywhere (except for well-grounded work-related or health reasons), all schools and universities were closed, gatherings in public places were prohibited, sporting events were suspended, all except vital businesses (supermarkets and pharmacies and related shops) were closed, and public transport was curtailed. Our survey was accessed by 1281 respondents, but 337 did not complete it and were consequently excluded from our analyses. Socio-demographic information was collected on respondents’ gender, age, number of children living at home, working or student status, and province of residence. The final sample comprised 944 participants (241 males) with a mean age of 37.24 years (SD = 14.50), and an age range between 18 and 81 years. In the sample as a whole, 35% had at least one child living at home with them, 49% were full-time workers, 21% were students, 9% worked part-time, 6% were unemployed, and 4% were retired. Respondents were living in 87 of Italy’s 106 provinces. Among the information requested, occupational changes were measured on a self-reported scale indicating how much respondents felt the pandemic had changed their job situation (from 1 = not at all, to 5 = drastically).

### Materials

*Values in Action Inventory of Strengths-120* (VIA-IS-120; Littman-Ovadia 2015; Peterson and Seligman 2004; Italian version provided by the VIA Institute). This tool consists of 120 items measuring character strengths (for a complete description, see the VIA Institute site at https://www.viacharacter.org/). There are 5 items for each strength, and answers are given on a 5-point Likert scale (1 = “not at all like me” to 5 = “very much like me”). For instance, curiosity is measured with items such as “I am always curious about the world” and perseverance is measured by items such as “I always complete what I begin”. The original measure showed a high internal consistency for every strength (Cronbach’s alpha range: 0.67–0.90, Peterson and Seligman 2004).

*Depression, Anxiety, and Stress Scales-21* (DASS-21, Lovibond and Lovibond 1995; validated in Italian by Bottesi et al. 2015). This scale comprises 21 items measuring three factors: depression, in terms of dysphoria, low self-esteem and absence of incentives (e.g. “I could not feel any positive emotion”); anxiety, in terms of somatic symptoms and fear responses (e.g. “I felt I was having a panic attack”); and stress, in terms of tension, high general arousal, impatience and irritability (e.g. “I felt stressed”). Respondents score on a 4-point Likert scale (0 = “never happened” to 3 “it happened almost every day”) how often they felt in such a way in the previous week. A total general distress score was calculated because it had proved highly reliable in the Italian validation study (Cronbach’s alpha = 0.90; Bottesi et al. 2015). After multiplying the score by 2, the cut-offs for moderate to extremely severe symptoms of anxiety, depression and stress are: >9, >13 and >18, respectively (Lovibond and Lovibond 1996).

*General Health Questionnaire-12* (GHQ-12, Goldberg 1978; validated in Italian by Giorgi et al. 2014). There are 12 items measuring general psychological health. Respondents indicate how often they felt as described during the previous two weeks (e.g. “Have you been able to concentrate on whatever you are doing?”) on a 4-point Likert scale (0 = “more than usual” to 4 “much less than usual”). A total score was calculated as in the Italian validation study, which showed a high internal consistency for the overall scale (Cronbach’s alpha = 0.85; Giorgi et al. 2014). The cut-off for lower than usual health is >12, as indicated by Goldberg et al. (1997).

*Self-efficacy measure for Covid-19* (SEC). Five questions were developed for the present study to measure self-efficacy beliefs about succeeding in various aspects of everyday life under quarantine (based on Bandura 2006): emotion regulation (e.g. “I feel I can manage the emotions I feel every day efficiently”); routine activity planning and completion (e.g. “I feel I can complete all my scheduled activities”); and interpersonal relationships (e.g. “I feel I can keep good relations with people important to me”). Answers are given on a 5-point Likert scale (1 = “not at all” to 5 = “completely”). A total score was calculated, given the high internal consistency (Cronbach’s alpha = 0.85; current sample).

### Procedure

All the questionnaires used in the survey were administered in the Italian version, were implemented in Qualtrics and took a mean 22 min to complete. A brief introduction to the study was sent to personal contacts, and posted on social media, with a link to the set of questionnaires. When participants opened the link, the consent form was presented, specifying the general aims and structure of the study. If they consented, they were first asked for various socio-demographic information, then they completed the survey in the following order: VIA-IS-120, DASS-21, GHQ-12 and SEC.

## Data Analysis

Before studying the relation between character strengths, symptoms of distress and self-efficacy, two preliminary steps were completed. First, we examined the prevalence of symptoms of psychological distress in our sample to describe the state of the population in Italy during lockdown. Then, the internal structure of the VIA-120 was examined, extracting the factor solution best fitting our data with the aid of a principal component analysis. This step was necessary to identify the second-order character strength effects as the theoretical 6-virtues structure proposed by Peterson and Seligman (2004) has not been confirmed, and several studies have since suggested different sets of second-order factors (e.g., three factors in McGrath 2015, and Shryack et al. 2010; or five in Höfer et al. 2019; Azañedo et al. 2017; Martínez-Martí and Ruch 2017; Littman-Ovadia 2015; McGrath 2014, and Ruch et al. 2010). Finally, to accomplish the main aim of our study, the associations of character strengths with the DASS-21, GHQ-12 and SEC were examined, in terms of both second-order and single character strengths, by means of a series of linear regression models.

Table 1 shows the means, standard deviations, Cronbach’s alpha and correlations for all the character strengths, DASS-21, GHQ-12 and SEC. All the measures revealed a good internal consistency, with Cronbach’s alpha ranging between 0.68 (for teamwork strength) and 0.96 (DASS-21). Correlations among each character strength are available in the supplementary material (Table S1).

**Table 1:** Means (M), standard deviations (SD), Cronbach’s alpha (α) and correlations among variables

	M	SD	α	DASS-21^a^	GHQ-12^b^	SEC^c^
*Character strengths*						
Appreciation of beauty	20.03	2.89	0.78	0.03	−0.05	0.11*
Bravery	18.31	3.20	0.80	−0.10*	−0.08	0.20*
Creativity	18.26	3.41	0.87	−0.10	−0.15*	0.25*
Curiosity	17.90	3.30	0.82	−0.22*	−0.24*	0.37*
Fairness	19.67	2.64	0.73	−0.01	0.01	0.09
Forgiveness	17.36	3.76	0.83	−0.21*	−0.10*	0.17*
Gratitude	18.88	3.35	0.84	−0.27*	−0.25*	0.34*
Honesty	21.31	2.33	0.78	−0.13*	−0.07	0.26*
Hope	17.50	3.56	0.78	−0.40*	−0.31*	0.43*
Humility	17.56	3.25	0.75	−0.06	−0.03	0.10*
Humor	18.63	3.34	0.81	−0.07	−0.14*	0.14*
Judgment	20.48	2.65	0.77	−0.02	−0.02	0.11*
Kindness	20.65	2.58	0.80	−0.06	−0.04	0.12*
Leadership	17.99	3.04	0.79	−0.06	−0.08	0.16*
Love	19.38	3.28	0.79	−0.23*	−0.17*	0.28*
Love of learning	17.58	3.61	0.78	−0.04	−0.10*	0.19*
Perseverance	18.88	3.41	0.87	−0.26*	−0.16*	0.37*
Perspective	17.75	3.20	0.82	−0.03	−0.04	0.13*
Prudence	17.71	3.29	0.79	−0.11*	−0.04	0.13*
Self-regulation	16.77	3.52	0.71	−0.22*	−0.17*	0.30*
Social intelligence	18.63	2.70	0.70	−0.09	−0.10*	0.19*
Spirituality	15.29	4.09	0.79	−0.24*	−0.22*	0.28*
Teamwork	18.31	2.70	0.68	−0.15*	−0.11*	0.16*
Zest	17.27	3.54	0.83	−0.35*	−0.31*	0.44*
DASS-21	15.10	10.70	0.96	1.00	0.54*	−0.52*
GHQ-12	17.01	4.90	0.79	0.54*	1.00	−0.58*
SEC	15.06	3.99	0.85	−0.52*	−0.58*	1.00

* = *p* < .002

For all tables: DASS-21 = Depression, Anxiety, and Stress Scales-21; GHQ-12 = General Health Questionnaire-12; SEC = Self-Efficacy measure for Covid-19. ^a^higher values indicate greater distress; ^b^higher values indicate worse mental health than usual; ^c^higher values indicate higher self-efficacy

## Results

### Distress Symptoms, Reliability and Correlation Among Variables

Scores obtained in the DASS-21 and GHQ-12 were calculated using standardized cut-offs to describe the severity of psychological distress in the population. On the three subscales of the DASS-21, 46% of participants presented moderate to extremely severe symptoms of depression, 40% presented moderate to extremely severe symptoms of stress, and 30% presented moderate to extremely severe symptoms of anxiety. From the results of the GHQ-12, 83% of participants reported a lower general state of health than usual.

## Factor Extraction: Principal Component Analysis

As suggested by Velicer et al. (2000), we first identified the number of components to extract from the data. Two criteria were used to do so: parallel analysis (PA, Horn 1965), and the minimum average partial (MAP) analysis (Velicer et al. 2000). Five eigenvalues were higher than 1 (7.78, 2.19, 1.83, 1.45, 1.09, 0.99), but both PA and MAP analysis indicated 4 as the best number of factors to extract from the data, so we extracted 4 factors. Five- and 6-factor solutions were also considered.

A principal component analysis was run to extract 4 factors using oblique rotation (promax), given that character factors hypothetically correlate with one another. Strengths were included in the factor in which they had the highest loading as long as the loading was at least 0.30. The results (see Table 2) showed that the four factors extracted were easy to interpret and composed by similar strengths than previous five-factor solutions, with the usually labelled emotional and intellectual factors being partially combined into a single factor, that we named openness, i.e. a positive attitude to exploring and open-mindedness, as represented by strengths such as creativity, curiosity, and bravery. Based on previously used names, the four factors were labeled: transcendence, interpersonal, openness, and restraint. These four factors jointly explained 55% of the variance, with a similar proportion each (i.e. 29%, 26%, 27%, and 19%, respectively).

**Table 2:** Four-factor solution for the VIA-IS 120

	Transcendence	Interpersonal	Openness	Restraint	h^2^
Hope	**0.77**	−00.10	**0.33**	−0.06	0.71
Spirituality	**0.74**	0.04	0.02	−0.17	0.52
Zest	**0.71**	−0.01	**0.47**	−0.20	0.78
Gratitude	**0.69**	0.15	0.11	−0.06	0.63
Perseverance	**0.64**	−0.20	0.16	0.25	0.54
Self-regulation	**0.54**	−0.17	−0.10	**0.41**	0.49
Love	**0.41**	0.22	0.15	−0.04	0.36
Fairness	−0.16	**0.86**	0.01	0.07	0.66
Kindness	0.01	**0.77**	0.16	−0.09	0.65
Teamwork	0.07	**0.73**	−0.08	−0.02	0.54
Leadership	−0.11	**0.66**	0.28	0.06	0.56
Humility	0.07	**0.55**	−**0.47**	0.25	0.51
Forgiveness	**0.39**	**0.43**	−0.29	−0.12	0.43
Appreciation of beauty	0.10	**0.38**	0.28	0.06	0.38
Creativity	0.14	−0.06	**0.71**	0.09	0.59
Bravery	0.13	−0.07	**0.68**	0.03	0.51
Curiosity	**0.50**	−0.10	**0.61**	−0.06	0.67
Humor	−0.05	**0.33**	**0.58**	−0.24	0.47
Social intelligence	0.03	**0.36**	**0.44**	0.14	0.51
Love of learning	0.18	−0.05	**0.40**	0.15	0.28
Prudence	−0.03	0.08	−0.27	**0.88**	0.76
Judgment	−0.16	−0.02	0.17	**0.84**	0.71
Perspective	−0.13	0.05	**0.34**	**0.63**	0.57
Honesty	0.20	0.22	0.15	**0.32**	0.41
Variance	0.16	0.14	0.15	0.10	

Bold = loading higher than .30

h^2^ = communality

Extracting the fifth and sixth component explained only a small percentage of additional variance (i.e. 4% and 5%, respectively). In addition, the five-factor structure, suggested by the number of eigenvalues higher than 1, and by other studies (e.g. Martínez-Martí and Ruch 2017; McGrath 2014; Ruch et al. 2010), also showed many cross-loadings, and one strength (honesty) not loading on any factor. Similarly, the six-factor structure showed a two-strengths factor and three strengths cross-loading on three of the six factors. The results of extracting the 5 and 6 components are available in the supplementary material (Table S2; Table S3).

In short, the second-order composition indicated that a four-factor structure was preferable as it emerged as the best solution using PA and MAP analyses. Moreover, we could not find any great psychometric, practical or theoretical improvement in considering the extractions with 5 or 6 factors. The four factors identified were therefore used in the subsequent analyses.

### Associations of Character Strengths with Psychological Distress, Mental Health and Self-efficacy

#### Associations of Second-Order Character Strengths Factors with Psychological Distress, Mental Health and Self-efficacy

Table 3 shows the correlations between the four factors and the DASS-21, GHQ-12 and SEC scores. Three linear regression models were run to measure the associations of the four factors (transcendence, interpersonal, openness and restraint) with the three psychological measures considered (DASS-21, GHQ-12 and SEC). In line with previous research on pandemics, demographic variables (such as age, gender, being a student, the day on which the participant took the survey, having at least one child living at home, and work-related changes) were added as possible factors explaining psychological distress and low mental health under quarantine (Nobles et al. 2020; Wang et al. 2020a, b). All the strength-related and outcome variables were standardized before running the regression analyses. Given our exploratory approach, the large number of participants, and the numerous dependent and independent variables, we took a conservative approach and considered as significant only the relations with an associated *p* value lower than 0.001.

**Table 3:** Correlations between second-order character strength factors (transcendence, interpersonal, openness and restraint), DASS-21, GHQ-12 and SEC

	1	2	3	4	5	6	7
1.Transcendence	1.00						
2. Interpersonal	0.51*	1.00					
3. Openness	0.63*	0.39*	1.00				
4. Restraint	0.33*	0.32*	0.32*	1.00			
5. DASS-21	−0.39*	−0.14*	−0.15*	−0.07	1.00		
6. GHQ-12	−0.32*	−0.09	−0.20*	−0.04	0.54*	1.00	
7. SEC	0.48*	0.20*	0.32*	0.15*	−0.52*	−0.58*	1.00

* = *p* < .001

The results of the three regression models (see Table 4) concerning the demographic variables identified more general distress (DASS-21) in women and respondents who reported more drastic work-related changes, and a lower self-efficacy (SEC) in women. None of the other demographic variables were significant, with *β* values lower than 0.22. The results for the associations of character strengths with the dependent variables showed significant and constant findings for transcendence across all three measures. This factor had the greatest associations with the DASS-21 (*β* = −0.48) and SEC (*β* = 0.48), followed by the GHQ-12 (*β* = −0.38), indicating that people well-endowed with transcendence strengths (e.g. hope, zest, gratitude) scored higher for general mental health, lower for psychological distress (fewer symptoms of depression, anxiety and stress), and higher for self-efficacy in coping with the lockdown situation. None of the associations of the other strengths with the three dependent variables were significant (*p* > 0.001) or large, as their *β* value never exceeded 0.08. The only exception was the second-order factor of openness, which showed a small but significant positive relation (*β* = 0.13) with the DASS-21, indicating that people scoring higher for openness (i.e. creativity, bravery, curiosity, humor, social intelligence, and love of learning) tended to experience more symptoms of depression, anxiety and stress under quarantine. Table 4 shows all the results of the three regression analyses.

**Table 4:** Results of the regressions models of strengths factors on DASS-21, GHQ-12 and SEC

	DASS-21		GHQ-12		SEC	
Predictor	*β*	CI	*β*	CI	*β*	CI
Age	−0.06	[−0.13, 0.01]	0.09	[0.02, 0.17]	0.02	[−0.05, 0.09]
Gender	−0.30*	[−0.44, −0.17]	−0.20	[−0.35, −0.07]	0.26*	[0.13, 0.39]
Student	0.11	[−0.06, 0.28]	0.23	[0.05, 0.40]	−0.10	[−0.26, 0.06]
Day of survey	−0.01	[−0.02, 0.00]	−0.01	[−0.01, 0.01]	0.01	[0.00, 0.02]
Work change	0.06*	[0.03, 0.10]	0.04	[0.00, 0.08]	−0.04	[−0.07, 0.00]
Having a child at home	−0.16	[−0.29, −0.03]	−0.01	[−0.14, 0.13]	0.03	[−0.09, 0.16]
Transcendence	−0.48*	[0.56, −0.40]	−0.38*	[−0.47, −0.29]	0.48*	[0.40, 0.56]
Interpersonal	0.05	[−0.02, 0.12]	0.08	[0.01, 0.15]	−0.06	[−0.13, 0.01]
Openness	0.13*	[0.06, 0.21]	−0.02	[−0.10, 0.06]	0.04	[−0.03, 0.12]
Restraint	0.02	[−0.05, 0.08]	0.07	[0.00, 0.14]	0.01	[−0.06, 0.06]
R^2^	0.22		0.13		0.26	

* = *p* < .001

*β* = beta value; CI = 95% confidence interval

Values refer to standardized variables

#### Associations of Single Character Strengths with Psychological Distress, Mental Health and Self-efficacy

To better understand the role of character strengths at a finer grain and shed light on the possible mechanisms behind the previous results, we ran again three multiple linear regressions, adding the 24 character strengths instead of the aggregated factors as predictors of the DASS-21, GHQ-12 and SEC scores. As done before, we added demographic covariates (i.e. age, gender, being a student, the day the participant took the survey, having at least one child living at home, and work-related changes). Bonferroni’s correction was applied to avoid type 1 errors. Given that 24 character strengths were added as predictors, we only considered the relations associated with a *p* value <0.002 as significant. Table 5 shows the results of the three regression models.

**Table 5:** Results of regressions with all 24 individual character strengths as predictors of psychological distress (DASS-21), mental health (GHQ-12) and self-efficacy (SEC)

	DASS-21		GHQ-12		SEC	
Predictor	*β*	CI	*β*	CI	*β*	CI
Age	−0.05	[−0.11, 0.00]	0.03	[0.00 0.06]	0.01	[0.00, 0.01]
Gender	−1.91	[−3.39, −0.44]	−0.59	[−1.32, 0.14]	0.22*	[0.08, 0.35]
Student	1.76	[0.01, 3.52]	1.20	[0.33, 2.07]	−0.09	[−0.25, 0.07]
Day of survey	−0.06	[−0.17, 0.03]	−0.02	[−0.07, 0.03]	0.01	[0.00, 0.02]
Work change	0.59	[0.20, 0.98]	0.15	[−0.04, 0.34]	−0.03	[−0.07, 0.00]
Having a child at home	−1.23	[−2.57, 0.09]	−0.11	[−0.55, 0.77]	0.01	[−0.12, 0.13]
Appreciation of beauty	0.56*	[0.29, 0.83]	0.13	[0.00, 0.27]	−0.09	[−0.16, −0.01]
Bravery	0.01	[−0.24, 0.25]	0.06	[−0.06, 0.18]	−0.03	[−0.10, 0.04]
Creativity	−0.01	[−0.25, 0.24]	−0.05	[−0.17, 0.07]	0.04	[−0.04, 0.12]
Curiosity	−0.01	[−0.31, 0.29]	−0.07	[−0.22, 0.08]	0.09	[−0.01, 0.18]
Fairness	0.25	[−0.07, 0.57]	0.19	[0.04, 0.35]	−0.04	[−0.11, 0.04]
Forgiveness	−0.30*	[−0.48, −0.11]	−0.02	[−.12, .07]	0.03	[−0.04, 0.09]
Gratitude	−0.01	[−0.28, 0.27]	−0.11	[−0.24, .03]	0.03	[−0.05, 0.12]
Honesty	−0.19	[−0.53, 0.14]	−0.01	[−0.18, 0.16]	0.09	[0.02, 0.16]
Hope	−0.82*	[−1.10, −0.53]	−0.17	[−0.31, −003]	0.13	[0.04, 0.23]
Humility	0.04	[−0.18, 0.26]	−0.07	[−0.18, 0.04]	0.05	[−0.01, 0.12]
Humor	0.10	[−0.12, 0.31]	−0.07	[−0.18, 0.03]	−0.02	[−0.08, 0.05]
Judgment	0.08	[−0.25, 0.42]	0.02	[−0.15, 0.18]	−0.02	[−0.10, 0.06]
Kindness	0.18	[−0.15, 0.52]	0.20	[0.03, 0.37]	−0.08	[−0.16, 0.00]
Leadership	0.17	[−0.10, 0.44]	−0.01	[−0.14, 0.13]	−0.02	[−0.09, 0.06]
Love	−0.36*	[−0.59, −0.14]	−0.07	[−0.18, 0.04]	0.11*	[0.04, 0.18]
Love of learning	0.13	[−0.06, 0.32]	−0.03	[−0.12, 0.07]	0.04	[−0.03, 0.10]
Perseverance	0.08	[−0.17, 0.33]	0.11	[−0.02, 0.23]	0.06	[−0.02, 0.14]
Perspective	0.18	[−0.06, 0.42]	0.08	[−0.04, 0.20]	−0.03	[−0.10, 0.04]
Prudence	−0.38*	[−0.65, −0.12]	−0.04	[−0.17, 0.09]	0.05	[−0.04, 0.13]
Self-regulation	−0.14	[−0.34, 0.06]	−0.09	[−0.19, 0.01]	0.08	[0.01, 0.14]
Social intelligence	0.24	[−0.05, 0.53]	0.05	[−0.09, 0.20]	−0.03	[−0.10, 0.04]
Spirituality	0.01	[−0.18, 0.19]	−0.10	[−0.19, 0.00]	0.02	[−0.05, 0.09]
Teamwork	−0.17	[−0.46, 0.13]	−0.08	[−0.22, 0.06]	−0.02	[−0.09, 0.06]
Zest	−0.58*	[−0.90, −0.25]	−**0.24°**	[−0.41, −0.08]	0.19*	[0.08, 0.30]
R^2^	0.29		0.17		0.30	

* = *p* < .002, °* p *= .003

*β* = beta value; CI = 95% confidence interval. Values refer to standardized variables

The results of the regression with the DASS-21 scores showed the associations of hope (*β* = −0.82), zest (*β* = −0.58) and love (*β* = −0.36), representing the transcendence strengths factor. Prudence (*β* = −0.38) and forgiveness (*β* = −0.30) were also negatively associated with distress symptoms, while appreciation of beauty showed a medium-to-large opposite association (*β* = 0.56). None of the other strengths and demographic variables showed any significant relation. As concerns the regression with SEC, we found only small associations of zest (*β* = 0.19) and love (*β* = 0.11)—again representing the transcendence strengths factor—and of gender, with females showing higher self-efficacy than males (*β* = 0.22). We only found a marginal significant association of zest (*β* = −0.24, *p* = 0.003) with GHQ-12 scores. Zest was the only strength significantly related with all the dependent measures considered, albeit only marginally in the regression on GHQ-12 scores.

## Discussion and Conclusion

The present study contributes to enlarge knowledge of the psychological impact of Covid-19 on the general population. It provides novel evidence of the associations between character strengths and psychological distress, and between character strengths and self-efficacy, in the context of a pandemic. We addressed the problem in the Italian population, one of the most badly-affected countries in the world (WHO 2020). First, it is important to mention that the levels of psychological distress exhibited by our respondents after a month under quarantine were similar to those reported in recently-published studies conducted both in Italy (e.g. Cellini et al. 2020; Moccia et al. 2020) and elsewhere (e.g. Wang et al. 2020b; Zhang et al. 2020), and indicate that the psychological impact of Covid-19 persists over time, as previously seen with SARS (Yeung and Fung 2007). Consistently with a four-week longitudinal study (Wang et al. 2020a, b), general distress levels in our sample were not affected by the day on which the survey was completed and remained stable over the three weeks during which our data were collected.

The structure of the 24 character strengths was analyzed and second-order components were found. Principal component analysis pointed to a four-factor as the best solution, in line with some previous studies (Brdar and Kashdan 2010; Macdonald et al. 2008). The strengths aggregated in much the same way as in previous reports, with some notable exceptions. For one, self-regulation and perseverance loaded in the transcendence instead of the restraint factor. Second, the openness factor considered in the present study is a combination of strengths usually belonging to the emotional factor (e.g. bravery, humor and social intelligence) and the intellectual factor (e.g. curiosity and love of learning). Following previous results that identified other number of factors (Littman-Ovadia 2015; McGrath 2014), five- and six-factor solutions were also examined, but the additional variance explained was negligible. Considering second-order factors has been suggested as an efficient way to examine the relations of character strengths with psychological outcomes (e.g. Martínez-Martí and Ruch 2017; Weber et al. 2013). Nevertheless, it has to be noted that factor composition varies across studies, and that the theoretical six-virtue structure proposed by Peterson and Seligman (2004) has not been empirically confirmed. These issues deserve to be better examined in future studies.

Turning to our main aim, we examined the associations between character strengths and both mental health (in general, and distress in particular) and Covid-19-related self-efficacy at both factor and single-strength levels. Regression models including second-order strength factors and demographic variables known to relate with psychological distress associated with pandemics (e.g. Brooks et al. 2020; Nobles et al. 2020) show that female gender and perception of work-related change due to Covid-19 were the only demographic variables significantly predicting the levels of general distress and self-efficacy. These findings are in line with previous reports regarding female gender and drastic work changes as predictors of psychological distress in people under quarantine (e.g. Huang and Zhao 2020; Shevlin et al. 2020).

As for second-order character strength factors, transcendence was the only factor showing strong negative associations with the indicators of psychological distress (DASS-21 and GHQ-12), and a positive association with Covid-19-related self-efficacy, in terms of people’s ability to manage their emotions, daily activities, and relations with others. These findings are in line with previous studies identifying transcendence strengths as the factor most associated with mental health in university students (Petkari and Ortiz-Tallo 2018), and young people living in stressful conditions (Shoshani and Slone 2016; Weber et al. 2013). The same relationship was also found in non-stressful conditions (Petkari and Ortiz-Tallo 2018), and in studies investigating single character strengths (Martínez-Martí and Ruch 2014). The same holds for general self-efficacy under stressful circumstances (Weber et al. 2013) and in non-stressful conditions (Martínez-Martí and Ruch 2017), as well as at single strength level (Ruch et al. 2014). All this suggests that the relationship between character strengths (particularly transcendence), psychological distress and self-efficacy is fundamental in both non-stressful and stressful conditions, a pandemic being among the latter. In our study, transcendence may refer to a sense of purpose beyond oneself, an orientation towards others (love), meaning (spirituality), positivity (hope, gratitude, zest), or self-sacrifice (persistence, self-regulation), akin to the transcendence virtue originally theorized by Peterson and Seligman (2004). Interestingly, subsequent regression analyses on the effect of single character strengths on the three dependent variables specifically identified the role of two transcendence strengths. These are zest, i.e. approaching life with energy and vitality, and love, i.e. appreciating being close to others. As single character strengths, they exhibited the strongest associations with both psychological distress and Covid-19-related self-efficacy. Hope (expecting the best for the future) was also significantly associated with psychological distress. Hope and zest reportedly have a strong effect on life satisfaction (see Bruna et al. 2019 meta-analysis), and are negatively associated with depression and anxiety (Niemiec 2013). They are also suggested to make individuals perceive less stress, and thus possibly contain the psychological distress experienced in challenging situations including being a caregiver (García-Castro et al. 2019), experiencing an earthquake (Duan and Guo 2015), living with a chronic illness like multiple sclerosis (Smedema 2020) or suffering from depression (Huta and Hawley 2010). Their role, together with that of love, may help to explain the positive relation of transcendence with the strain of lockdown in a pandemic. Regression models with single character strengths as predictors also identified two other strengths, forgiveness and prudence, as being significantly and negatively associated with psychological distress. Following Niemiec’s (2019) conceptualization, these strengths may have both a reappraisal and a resilience function in a lockdown: they can support individuals’ positive reframing of the situation, making them better able to appreciate smaller pleasures. Such a positive attitude is usually associated with better psychological outcomes, in both normal and exceptional conditions (Niemiec 2013; Shoshani and Slone 2016). As lockdown measures become less stringent, transcendence may also support people’s resilience, possibly helping them to experience less psychological distress in the future as well (Martínez-Martí and Ruch 2017).

Contrary to our expectations, the restraint factor was not associated with psychological distress or Covid-19-related self-efficacy. As mentioned earlier, the restraint factor identified in our study differed from that of other studies, and did not include perseverance and self-regulation, which had previously been found related with both psychological distress and self-efficacy (e.g. Weber et al. 2013). This may partly explain why the associations between this factor and the dependent variables were not significant in the present work.

Surprisingly, when the other strengths factors were considered in the analysis, the openness factor showed a significant positive relation with psychological distress, as measured by the DASS-21. Though the relation was small, and not seen for the other dependent variables, this finding goes against our expectations from a theoretical standpoint (Niemiec 2019). In this respect, it is worth noting that openness (which comprises creativity, bravery, curiosity, humor, social intelligence, and love of learning) refers to a general disposition to seek and create stimuli and emotions to make life fulfilling. Such a disposition might be curtailed by the inability to express these feelings due to limitations on an individual’s interpersonal relationships and activities under lockdown. This may also help to explain the positive association between appreciation of beauty and psychological distress that emerged from the regression analyses taking single character strengths into account. Similar findings were reported in a study on Israeli adolescents exposed to prolonged war and terrorism (Shoshani and Slone 2016): intellectual strengths, such as curiosity, creativity and love of learning, revealed a small, but significant positive relationship (*β* = 0.13) with psychological distress, as measured by the Global Severity Index. The study’s authors suggested that, in highly-stressful situations, seeking information and enjoying knowledge could have a detrimental effect, exacerbating psychological distress instead of alleviating it. Studies on media exposure after terrorist attacks show, for instance, that media consumption is associated with post-traumatic stress symptoms (Ahern et al. 2002), and anxiety (Slone and Shoshani 2010). A study on the impact of Covid-19 (Huang and Zhao 2020) also found that the amount of time spent each day focusing on news about the infection was significantly associated with anxiety in Chinese general population.

Further studies are needed to better understand the association of openness factor with psychological distress and to investigate whether character strengths levels change following lockdown, as seen after September 11 (Peterson and Seligman 2003). Our study, in fact, relies on data collected during the pandemic and cannot ensure that strengths levels are representative of before-pandemic strengths, this being a limitation of our research. Our results indicate that transcendence as a second-order factor (and zest and love as single strengths) have a clear relation with self-perceived level of distress and pandemic-related self-efficacy in times of Covid-19. This relation seems to be detectable in stressful as well as non-stressful conditions (using a qualitative comparison at least; Martínez-Martí and Ruch 2017). That said, we cannot say for sure that these effects are peculiar to the pandemic because no pre- or post- pandemic measurements of character strengths have been carried out. Future studies investigating character strengths post-pandemic may shed light on any character growth, and correlate during-pandemic strength levels with post-pandemic psychological distress. Another limitation of our work concerns gender. Since our sample was not balanced by gender our results on females displaying higher psychological distress than males could be biased, and therefore not necessarily supporting previous reports. For instance, we found a significant role for appreciation of beauty in relation to distress, and for love in relation to both distress and self-efficacy. This finding may be influenced by the greater prevalence of female participants, who may have higher levels of these strengths than males, as reported by Heintz et al. (2019). Their meta-analysis showed that gender differences are less pronounced in adults (especially in 21- to 24-year-olds) than in children and adolescents, and in short rather than in standard VIA measures. Therefore, gender biases in our study (conducted on adults and using a shorter measure) might have been less pronounced.

In conclusion, this study examines the associations of character strengths with Covid-19-related psychological distress and self-efficacy under lockdown in such a severely-affected population as the Italian one. The strengths most associated with our dependent variables were transcendence at second-order level, and zest and love at single strengths’ level: these strengths seem to be associated with better mental health (i.e. lower levels of distress) and higher self-efficacy regarding how best to approach the situation brought on by the pandemic. Such personal characteristics may have been especially relevant in a situation of prolonged quarantine, when all citizens—whether they were experiencing symptoms or not—were asked to stay at home and self-isolate to prevent the virus from spreading.

In the light of our results, the Italian Covid-19 slogan “*andrà tutto bene”* (i.e. everything is going to be alright) disseminated by the media seems to express the right attitude, helping people to deal with the lockdown and engage in positive behaviors and hopeful thoughts, which might ultimately sustain their psychological response to these stressful circumstances.

## Electronic supplementary material

Below is the link to the electronic supplementary material.

Supplementary file1 (DOCX 20 kb)

## Data Availability

Data are available on Figshare. https://doi.org/10.6084/m9.figshare.12366494
