# Supplementary material for: “Andrà tutto bene”: Associations Between Character Strengths, Psychological Distress and Self-efficacy During Covid-19 Lockdown
Source: J Happiness Stud. 2020 Oct 13;22(5):2255–74. doi: 10.1007/s10902-020-00321-w (PMC7553371; doi:10.1007/s10902-020-00321-w)
Supplement: Supplementary file 1 — Supplementary file1 (DOCX 20 kb) [file 10902_2020_321_MOESM1_ESM.docx]

Supplementary material

Table S1. Bivariate correlations between each character strength, the Depression Anxiety and Stress Scale-21 (DASS-21), the General Health Questionnaire-12 (GHQ-12), and the Self-Efficacy measure for Covid-19 (SEC).

|  | 1. | 2. | 3. | 4. | 5. | 6. | 7. | 8. | 9. | 10. | 11. | 12. | 13. | 14. | 15. | 16. | 17. | 18. | 19. | 20. | 21. | 22. | 23. |
| --- | --- | --- | --- | --- | --- | --- | --- | --- | --- | --- | --- | --- | --- | --- | --- | --- | --- | --- | --- | --- | --- | --- | --- |
| 1.Appreciation of beauty | - |  |  |  |  |  |  |  |  |  |  |  |  |  |  |  |  |  |  |  |  |  |  |
| 2.Bravery | .25 | - |  |  |  |  |  |  |  |  |  |  |  |  |  |  |  |  |  |  |  |  |  |
| 3.Creativity | .37 | .43 | - |  |  |  |  |  |  |  |  |  |  |  |  |  |  |  |  |  |  |  |  |
| 4.Curiosity | .38 | .42 | .63 | - |  |  |  |  |  |  |  |  |  |  |  |  |  |  |  |  |  |  |  |
| 5.Fairness | .38 | .17 | .20 | .25 | - |  |  |  |  |  |  |  |  |  |  |  |  |  |  |  |  |  |  |
| 6.Forgiveness | .19 | -.02 | .07 | .20 | .38 | - |  |  |  |  |  |  |  |  |  |  |  |  |  |  |  |  |  |
| 7.Gratitude | .46 | .26 | .28 | .46 | .33 | .30 | - |  |  |  |  |  |  |  |  |  |  |  |  |  |  |  |  |
| 8.Honesty | .34 | .36 | .30 | .32 | .36 | .16 | .36 | - |  |  |  |  |  |  |  |  |  |  |  |  |  |  |  |
| 9.Hope | .32 | .37 | .42 | .59 | .24 | .29 | .65 | .30 | - |  |  |  |  |  |  |  |  |  |  |  |  |  |  |
| 10.Humility | .24 | -.05 | -.04 | -.03 | .34 | .30 | .22 | .23 | .08 | - |  |  |  |  |  |  |  |  |  |  |  |  |  |
| 11.Humor | .26 | .34 | .34 | .36 | .21 | .07 | .27 | .18 | .33 | .01 | - |  |  |  |  |  |  |  |  |  |  |  |  |
| 12.Judgment | .26 | .22 | .28 | .24 | .24 | .08 | .19 | .32 | .21 | .12 | .06 | - |  |  |  |  |  |  |  |  |  |  |  |
| 13.Kindness | .45 | .23 | .27 | .33 | .56 | .32 | .46 | .43 | .34 | .28 | .36 | .18 | - |  |  |  |  |  |  |  |  |  |  |
| 14.Leadership | .30 | .33 | .30 | .34 | .56 | .19 | .32 | .36 | .33 | .22 | .31 | .21 | .45 | - |  |  |  |  |  |  |  |  |  |
| 15.Love | .33 | .23 | .21 | .34 | .25 | .17 | .49 | .32 | .45 | .13 | .24 | .16 | .41 | .33 | - |  |  |  |  |  |  |  |  |
| 16.Love of learning | .32 | .30 | .38 | .42 | .22 | .16 | .26 | .23 | .30 | .05 | .16 | .28 | .18 | .21 | .16 | - |  |  |  |  |  |  |  |
| 17.Perseverance | .17 | .41 | .28 | .44 | .20 | .15 | .39 | .51 | .50 | .16 | .15 | .25 | .22 | .32 | .30 | .24 | - |  |  |  |  |  |  |
| 18.Perspective | .26 | .26 | .35 | .29 | .21 | .06 | .26 | .28 | .30 | .10 | .19 | .52 | .24 | .31 | .22 | .20 | .24 | - |  |  |  |  |  |
| 19.Prudence | .14 | -.06 | .06 | .05 | .23 | .11 | .18 | .28 | .14 | .34 | -.05 | .60 | .15 | .18 | .12 | .07 | .26 | .43 | - |  |  |  |  |
| 20.Self-regulation | .20 | .16 | .20 | .26 | .18 | .17 | .33 | .32 | .36 | .24 | .10 | .25 | .17 | .22 | .28 | .21 | .41 | .24 | .37 | - |  |  |  |
| 21.Social intelligence | .36 | .36 | .39 | .39 | .34 | .16 | .38 | .34 | .44 | .13 | .37 | .28 | .48 | .46 | .37 | .24 | .33 | .43 | .20 | .24 | - |  |  |
| 22.Spirituality | .32 | .21 | .25 | .38 | .23 | .33 | .51 | .28 | .48 | .13 | .12 | .06 | .33 | .24 | .29 | .20 | .35 | .16 | .07 | .25 | .30 | - |  |
| 23.Teamwork | .22 | .12 | .15 | .24 | .50 | .31 | .34 | .31 | .29 | .33 | .21 | .15 | .47 | .51 | .31 | .13 | .26 | .15 | .23 | .18 | .33 | .28 | - |
| 24.Zest | .36 | .45 | .49 | .71 | .26 | .24 | .61 | .35 | .75 | .06 | .45 | .12 | .41 | .38 | .44 | .31 | .54 | .25 | .05 | .32 | .46 | .49 | .35 |

All correlations with |r| ≥ .11 are significant at the .001 level.

Table S2. Character strengths: five-factor solution

|  | F1 | F2 | F3 | F4 | F5 | h^2^ |
| --- | --- | --- | --- | --- | --- | --- |
| Hope | **.79** | .10 | .06 | .15 | .18 | .71 |
| Perseverance | **.78** | .15 | .19 | .20 | .23 | .68 |
| Zest | **.75** | .00 | .21 | **.32** | .15 | .79 |
| Spirituality | **.69** | .02 | .15 | .16 | .19 | .54 |
| Gratitude | **.66** | .14 | .05 | .05 | .18 | .64 |
| Self-regulation | **.60** | .15 | **.39** | .12 | .09 | .51 |
| Curiosity | **.49** | .12 | .04 | **.34** | **.44** | .70 |
| Love | **.45** | .25 | .07 | .16 | .07 | .39 |
| Honesty | .29 | .26 | .28 | .21 | .11 | .47 |
| Fairness | .21 | **.85** | .08 | .02 | .11 | .66 |
| Kindness | .02 | **.77** | .08 | .15 | .10 | .65 |
| Teamwork | .09 | **.76** | .05 | .04 | .20 | .58 |
| Leadership | .05 | **.70** | .03 | **.39** | .11 | .63 |
| Humility | .02 | **.55** | .25 | **.41** | .12 | .52 |
| Prudence | .00 | .08 | **.86** | .25 | .03 | .76 |
| Judgment | .16 | .04 | **.86** | .04 | **.33** | .74 |
| Perspective | .09 | .04 | **.63** | .25 | .25 | .57 |
| Bravery | .24 | .03 | .01 | **.66** | .06 | .57 |
| Humor | .01 | **.35** | .26 | **.57** | .10 | .49 |
| Forgiveness | .24 | **.37** | .07 | **.47** | .27 | .56 |
| Social intelligence | .08 | **.38** | .12 | **.42** | .10 | .53 |
| Love of learning | .07 | .12 | .22 | .06 | **.66** | .50 |
| Appreciation of beauty | .02 | **.33** | .12 | .02 | **.55** | .54 |
| Creativity | .13 | .09 | .12 | **.45** | **.53** | .63 |
| Variance | .17 | .14 | .10 | .10 | .08 |  |

Note. Bold = loading higher than .30; h^2^ = communality.

Table S3. Character strengths: six-factor solution

|  | F1 | F2 | F3 | F4 | F5 | F6 | h^2^ |
| --- | --- | --- | --- | --- | --- | --- | --- |
| Gratitude | **.79** | .04 | .08 | .04 | -.04 | -.01 | .70 |
| Hope | **.78** | -.17 | .14 | -.00 | .05 | .17 | .74 |
| Spirituality | **.71** | .05 | -.13 | -.14 | .10 | .07 | .55 |
| Zest | **.68** | -.07 | .28 | -.17 | .10 | .23 | .79 |
| Love | **.60** | .02 | **.36** | .08 | **-.32** | -.01 | .53 |
| Fairness | -.20 | **.86** | .21 | -.01 | .16 | -.02 | .71 |
| Teamwork | .03 | **.70** | .23 | -.10 | -.17 | .16 | .59 |
| Humility | -.01 | **.64** | -.26 | .15 | -.09 | .09 | .53 |
| Leadership | -.17 | **.61** | **.50** | -.03 | -.03 | .21 | .65 |
| Kindness | .15 | **.60** | **.44** | -.02 | -.04 | -.16 | .66 |
| Forgiveness | **.35** | **.50** | **-.34** | -.13 | .23 | -.12 | .57 |
| Humor | .13 | .09 | **.73** | -.12 | -.04 | -.12 | .53 |
| Social intelligence | .18 | .15 | **.57** | .23 | -.06 | -.03 | .58 |
| Prudence | -.02 | .08 | -.20 | **.85** | -.13 | .11 | .78 |
| Judgment | -.16 | -.04 | -.01 | **.84** | .26 | .01 | .74 |
| Perspective | .04 | -.14 | **.33** | **.74** | .03 | -.11 | .67 |
| Love of learning | -.06 | .11 | -.18 | .06 | **.84** | .06 | .65 |
| Creativity | .08 | -.09 | **.30** | .10 | **.56** | .03 | .64 |
| Curiosity | **.41** | -.10 | .20 | -.06 | **.46** | .14 | .71 |
| Appreciation of beauty | .23 | .27 | .17 | .18 | **.37** | **-.32** | .55 |
| Perseverance | .28 | -.01 | -.07 | .01 | .03 | **.77** | .77 |
| Honesty | -.05 | **.34** | .09 | .12 | .09 | **.50** | .56 |
| Bravery | -.09 | -.02 | **.43** | -.11 | .28 | **.44** | .64 |
| Self-regulation | **.36** | -.05 | -.25 | **.30** | -.04 | **.44** | .51 |
| Variance | .15 | .12 | .11 | .10 | .08 | .08 |  |

Note. Bold = loading higher than .30; h^2^ = communality.
